# Membrane-Accelerated Amyloid-*β* Aggregation and Formation of Cross-*β* Sheets

**DOI:** 10.3390/membranes7030049

**Published:** 2017-08-31

**Authors:** Adree Khondker, Richard J. Alsop, Maikel C. Rheinstädter

**Affiliations:** Department of Physics and Astronomy, McMaster University, 1280 Main Street West, Hamilton, ON L8S 4M1, Canada; khondkea@mcmaster.ca (A.K.); alsoprj@mcmaster.ca (R.J.A.)

**Keywords:** Alzheimer’s disease, amyloid-*β*, hydrophobic fragment, cross-*β* sheet

## Abstract

Amyloid-β aggregates play a causative role in Alzheimer’s disease. These aggregates are a product of the physical environment provided by the basic neuronal membrane, composed of a lipid bilayer. The intrinsic properties of the lipid bilayer allow amyloid-β peptides to nucleate and form well-ordered cross-β sheets within the membrane. Here, we correlate the aggregation of the hydrophobic fragment of the amyloid-β protein, $A\beta_{25-35}$, with the hydrophobicity, fluidity, and charge density of a lipid bilayer. We summarize recent biophysical studies of model membranes and relate these to the process of aggregation in physiological systems.

## 1. The Amyloid State in Alzheimer’s Disease

Degenerative diseases of the human brain have long been viewed among the most puzzling and difficult problems in biomedical sciences. As researchers have begun to uncover the mechanistic underpinnings of neurodegenerative diseases, it has become increasingly apparent that such many diseases have both biochemical and biophysical roots [1,2,3,4]. Many of these diseases have been characterized by small deposits of extracellular filaments, which build up in the neuronal tissue [5]. As more sophisticated structural analyses procedures were developed, these filaments were found to be composed of thousands of monomeric fragments. Such fragments were soon classified as amyloids based on their distinctive scattering pattern [6].

Many proteins, especially misfolded prions, enter the so-called amyloid state when they form elongated fibers with “spiny” β-sheets [2]. Alzheimer’s disease is characterized by the aggregation of insoluble fibrillary amyloid-β (Aβ) peptides in the extracellular space of neural tissue, resulting in neural atrophy [1,7,8]. In healthy individuals, the amyloid precursor protein (APP) is an integral membrane protein, which is thought to be necessary for synapse formation. The 42-residue transmembrane fragment of APP, which spans the external leaflet of the membrane, is called Aβ. APP is cleaved to release both cytoplasmic and extracellular domains, which harbor both intracellular and extracellular function. However, the improper cleavage of the protein leads to the release of an elongated cytoplasmic domain, and a truncated inter-membrane domain. This inter-membrane domain undergoes further proteolysis to produce A$\beta_{1-42}$. The predominant Aβ species formed from improper cleavage are A$\beta_{1-40}$ and A$\beta_{1-42}$; both of which contain the transmembrane A$\beta_{25-35}$ domain [9,10,11,12,13].

There are three key suggested mechanisms by which the amyloid state induces physiological damage in the neuronal milieu in Alzheimer’s disease. First, the formation of Aβ oligomers and aggregates can promote the formation of radical oxygen species, which can promote the activation of caspases and thus neuronal cell death. Second, oligomers of Aβ can promote the activation of toll-like receptors and promote local inflammation leaving residual toxic damage. Finally, the formation of cross-β sheets in the membrane can facilitate the formation of β-barrels or channel pores by intercalating, which leads to increased Ca${}^{2+}$ influx and subsequent activation of caspases. Current literature suggests that a combination of all three mechanisms is likely to occur simultaneously [14,15]. The actual mechanism may also depend on the Aβ fragment: while A$\beta_{1-40}$ were observed to aggregate into amyloid fibrils, A$\beta_{1-42}$ assembled into oligomers that inserted into lipid bilayers as well-defined β-barrel channels [16].

Indeed, these mechanisms call upon the aggregation and subsequent oligomerization of the improperly processed Aβ. In this review, we will illustrate the role of the lipid bilayer in promoting these initial stages in the pathogenesis of Alzheimer’s disease in both the A$\beta_{1-42}$ and A$\beta_{25-35}$ domains of the peptide. We will discuss the current state of literature on the structure of Aβ to compare mechanisms leading to peptide oligomerization in solution or on a membrane support. The emphasis of this review will be to explain the relationships between membrane properties and the structure of the main Aβ proteins in monomeric and polymeric forms.

## 2. Aggregation of Amyloid-β on Lipid Membranes

**Figure 1:**
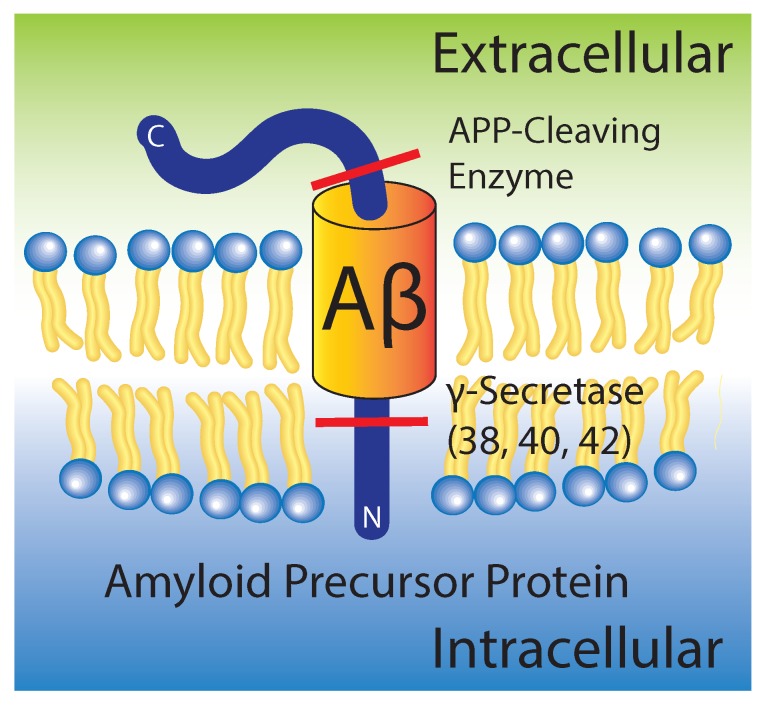
Schematic of amyloid precursor protein in its “original” position within the membrane prior to cleavage and release of intra- and extracellular fragments. Amyloid-β (Aβ) is initially in the external leaflet of the membrane, where it only slightly perturbs the membrane.

The major pathological hallmark of Alzheimer’s disease is the formation of protein aggregates or “plaques” in neural tissue. These aggregates are composed of oligomers of A$\beta_{1-40/42}$ formed from the improper proteolytic cleavage and clearance of the amyloid precursor protein by β- and γ-secretases, as shown in Figure 1.

**Figure 2:**
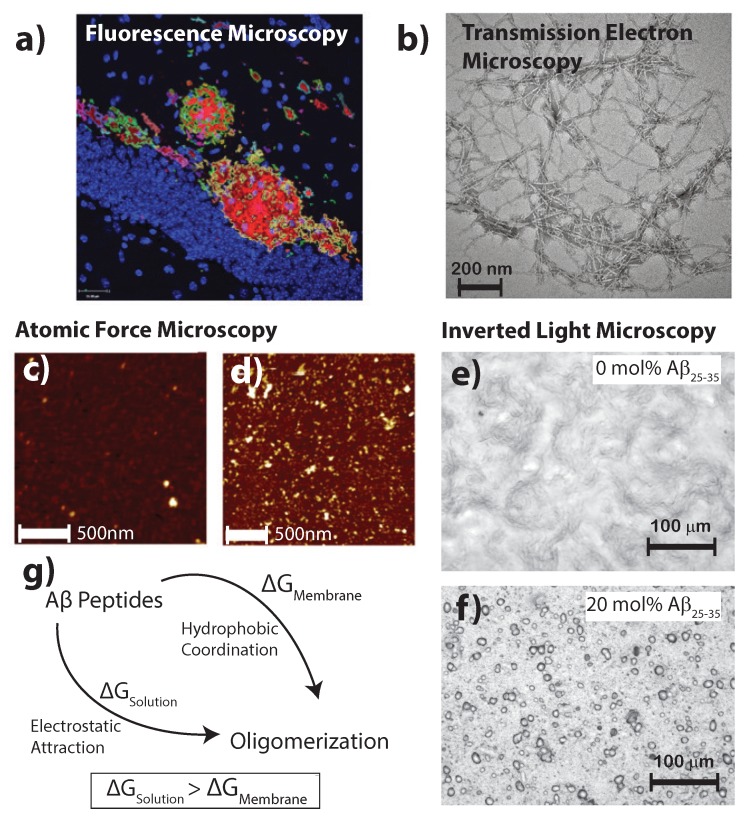
(**a**) Fluorescent secondary antibodies to the Aβ plaques in mouse models of Alzheimer’s disease are shown as per Fisher et al. [24] (copyright PLoS ONE, 2010); (**b**) Transmission electron microscopy of crystalized Aβ photofibrils in solution from Chen et al. [25] (copyright PLoS ONE, 2012); Atomic force microscopy images in liquid shows $A\beta_{1-42}$ in healthy (**c**) and diseased (**d**) membrane models; bars correspond to 500 and 1000 nm, respectively, and the image adapted from the preprint by Drolle et al. [19] (copyright arXiv, 2017); Optical microcopy images of (**e**) a pure 1-palmitoyl-2-oleoyl-sn-glycero-3-phosphocholine (POPC)/1,2-ditetradecanoyl-sn-glycero-3-phospho-L-serine (DMPS) membrane and (**f**) POPC/DMPS + 20 mol % A$\beta_{25-35}$. While the pure lipid matrix shows a smooth surface, inclusions were observed at peptide concentrations of 10 and 20 mol % [21] (copyright Royal Society of Chemistry, 2016); (**g**) The consenses of literature suggests the formation of Aβ aggregates is more favorable in the presence of a membrane than in pure solution.

Tau is a component that is often intercalated with the surface of the aggregates with its own distinctive characteristics, as detailed, for instance, in Spires-Jones and Hyman [17]. Although aggregates often manifest in the formation of visible plaques associated with the pathology of Alzheimer’s disease, the formation of these plaques has been widely characterized with light, confocal, electron and atomic force microscopy (AFM), as depicted in Figure 2. Clinically, ∼100 plaques/mm${}^{2}$ are observed [18]. AFM experiments observe a depression in the membrane by 5 Å in samples containing Aβ aggregates. These plaques were found to reduce the surface electrostatic potential by 7-fold in the diseased state [19]. In solution, the majority of oligomers have heights of 1.5 – 2.5 nm [20]. However, plaques of pure A$\beta_{25-35}$ in supported lipid membranes were found to have a diameter of ∼11 μm [21]. If the peptide conformation allows for β-sheets to form on length scales of ∼5 Å, a very high density of peptides in these plaques can be expected, which would have a drastic effect on the membrane surface. Together, these studies have given rise to characterization of these plaques through mathematical modeling while providing a quantification for nanoscopic structural features of the A$\beta_{1-42}$ peptide through the amyloid cascade hypothesis [22,23].

Current models of aggregation include: (1) the Bell-Evans Model; (2) the Dudko-Hummer-Szabo Model; and (3) the Friddle-De Yoreo model, as compared by Leonenko and colleagues in 2014. The three models allow for the calculation of free energy of aggregation ($\Delta G_{Aggregation}$) as a function of the forces holding together aggregates [26]. It is important to note that these parameters are related to the stability of an aggregate of A$\beta_{1-42}$, which depends on the presence of a membrane support. Indeed, the cell membrane offers a unique interface for the stabilization and modulation of protein dynamics and aggregation. Thus, the atomistic interactions between Aβ are likely modulated by the presence of a membrane and can affect the kinetics of aggregation.

**Figure 3:**
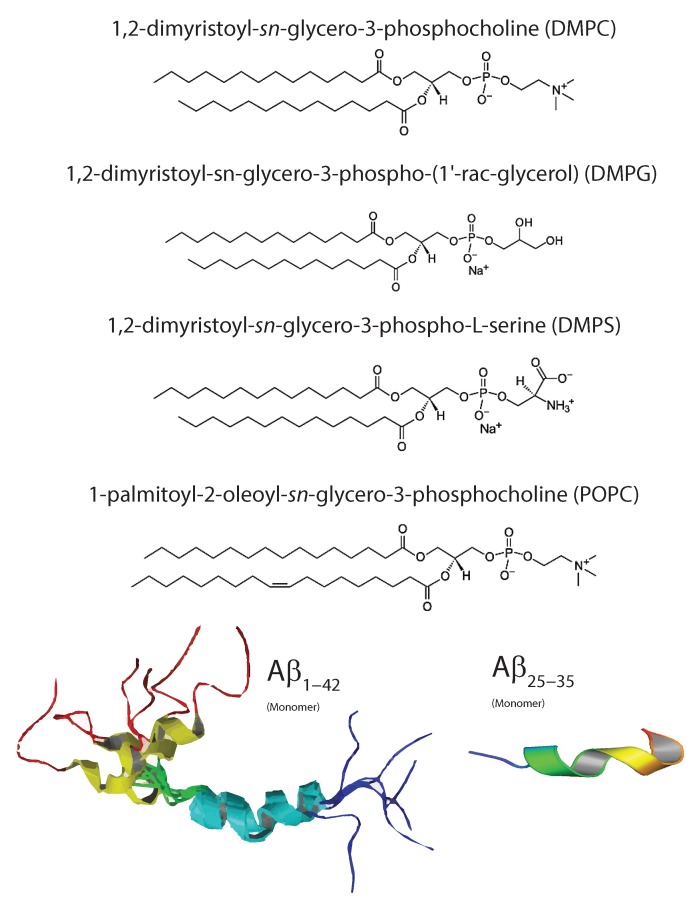
Typical phospholipids used in studies involving membrane-mediated interactions with Aβ include: 1,2-dimyristoyl-sn-glycero-3-phosphocholine (DMPC) which is a fully saturated and good model of bilayer properties; 1,2-dimyristoyl-sn-glycero-3-phosphorylglycerol (DMPG) which allows for modification of bilayer surface charge; 1,2-dimyristoyl-sn-glycero-3-phospho-l-serine (DMPS) which is an anionic lipid for understanding electrostatic surface potential; and 1-palmitoyl-2-oleoyl-sn-glycero-3-phosphocholine (POPC) which is the standard model membrane for eukaryotic membranes due to its half-saturated, half-unsaturated organization and fluidity in bilayer systems. Monomeric forms of the A$\beta_{1-42}$ and A$\beta_{25-35}$ are shown from solution NMR (PDB structures 1Z0Q and 1QWP).

The plasma membrane separates the interior of the cell from the external environment and is composed of proteins, small molecules, and of course, the phospholipid bilayer. Although physiological cell membranes are composed of many different lipids, uni- or multi-component model membranes can mimic the properties of the cellular membrane milieu. For example, model membranes composed of the lipid 1-palmitoyl-2-oleoyl-sn-glycero-3-phosphocholine (POPC) mimic the acyl tail saturation, membrane fluidity, and the typical hydrodynamic diameter of typical cell membranes. At small concentrations of the anionic lipid 1,2-ditetradecanoyl-sn-glycero-3-phospho-L-serine (DMPS), the anionic charge of the bilayer surface can be modeled. Figure 3 depicts the molecular solution structure of lipids, A$\beta_{1-42}$ and A$\beta_{25-35}$ as of PDB references 1Z0Q and 1QWP, respectively. The A$\beta_{1-42}$, and the A$\beta_{25-35}$ structure depicted was resolved by solution-NMR with 30 and 20 conformers calculated [27,28]. For further information on the construction of model membranes, please see [29].

Fibril formation of $A\beta_{1-42}$ on model supported lipid bilayers showed progressive accumulation of oligomers and short photofibrils. The presence of positively charged and fluid lipid bilayers was found to interfere with aggregation of A$\beta_{1-42}$ [30]. The long-range electrostatic interactions that promote aggregation were identified, and used in Monte Carlo simulations which led to the formation of aggregates in a lipid interface within 40 min [21].

The hydrophobic A$\beta_{25-35}$ domain can independently have neurotoxic effects [31], but is more often accredited for being essential in the membrane anchoring and interaction of the full A$\beta_{1-42}$ with the cell membrane leading to aggregation [32]. The physical basis for the aggregation of Aβ has been explored extensively; however, the physical basis for A$\beta_{25-35}$ membrane-mediated effects remains debated.

Aβ aggregates consist of thousands of the monomeric amyloid proteins attempting to minimize the final free energy by utilizing the lipid membrane. Indeed, while the characterization and growth of these plaques is well understood at a macroscopic level, the mechanisms that initiate this aggregation from the monomeric form of Aβ remain elusive [33]. The two mechanisms proposed by Bokvist and colleagues suggest there is: (1) electrostatic attraction between the Aβ and lipid head groups; and (2) hydrophobically-driven peptide insertion [34]. Recent literature suggests that there is a combination of these processes that drives the aggregation of Aβ in the presence of lipid bilayers.

## 3. Molecular Structure of Amyloid-β

In 1959, the first unbranched fibrils were reported in electron micrographs of diseased tissues [35] and nine years would pass before X-ray diffraction would identify the characteristic cross-β structure of amyloid fibrils [36]. Today, the advent of numerous structure-determining techniques, such as nuclear magnetic resonance (NMR), X-ray and neutron diffraction, have elucidated the molecular structure of A$\beta_{1-42}$ and A$\beta_{25-35}$. The fibril core consists of a dimer of A$\beta_{1-42}$ molecules, each containing four β-strands in a S-shaped amyloid fold [37].

X-ray diffraction is a prominent analytical technique used for the identification of the intrinsic periodicities in molecular structure. Incident X-rays diffract from repeating features within a sample, and the parallel diffracted waves will be shifted in phase with respect to the distance between the features of the sample based on Bragg’s law. According to Bragg’s law, the most basic distance between two lattices, *d*, can be calculated from

(1) nλ=2dsinθ

where *n* is some integer, λ is wavelength, and θ is the scattering angle.

**Figure 4:**
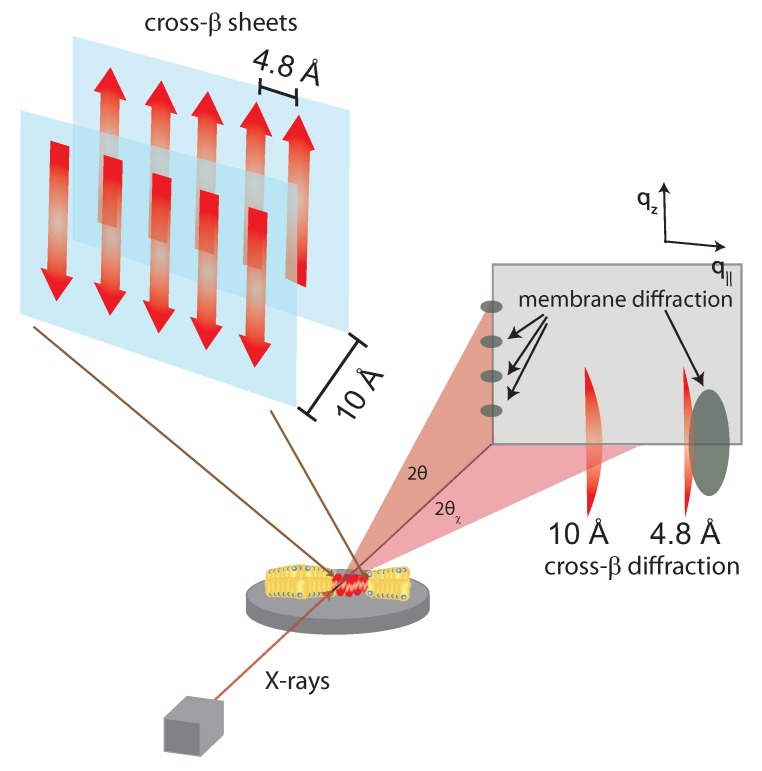
Structure of a cross-β sheet. The 4.8 Å distance corresponds to chain distances within a sheet while the ∼10 Å distance is the distance between antiparallel sheets. When cross-β sheets form in the presence of membranes, additional signals occur related to membrane stacking and packing of the acyl-tails in the hydrophobic membrane core [21].

Initial powder scattering of Aβ showed two diffuse bands corresponding to distances of 4.8 Å and 9.8 Å, respectively. Such signals agree with the expected β-strands running in-register to one another, as shown in Figure 4. For model-building results from samples of Aβ fibers, artificial periodicity must be introduced into the sample preparation in order to attain quantifiable scattering signals. Aβ can be prepared in a stretch frame or with thin-film diffraction using a cryoloop preparation of solvent polypeptides to result in in-plane periodicity of the fibers [38,39]. The X-ray diffraction pattern of cross-β sheets consists of two signals corresponding to the 4.8 and ∼10 Å periodicities. When the cross-β sheets form in the presence of membranes, additional signals occur corresponding to the lamellar stacking of the bilayers and the acyl-tail correlation peak.

Although residues 1–17 are disordered, residues 18–42 in A$\beta_{1-42}$ form a β-turn motif that contains parallel in-register β-sheets formed by residues 18–26 ($\beta_{1}$) and 31–42 ($\beta_{2}$) [40]. In the fragment A$\beta_{25-35}$, the β-sheet is formed from hydrogen bonding between amide residues from neighboring peptides. The formation of these lateral hydrogen bonds can be accelerated by the stability of the water-hydrophobic interface provided by the cell membrane. It was also suggested that the antiparallel β-sheets are zipped together by adjacent π-bonding between adjacent phenylalanine rings and salt-bridges between charge pairs (glutamate-lysine) [39,41].

There is a particular importance in the interaction of Aβ and the cell membrane as it can promote non-native and toxic structural configurations of the peptide. Specifically, X-ray and neutron diffraction studies of A$\beta_{1-42}$ and A$\beta_{25-35}$ in multilamellar stacks of lipid bilayers on a solid support have been critical in understanding the membrane-bound structure of the peptide and the strong dependence of the membrane-mediated elastic interaction of the peptides.

**Figure 5:**
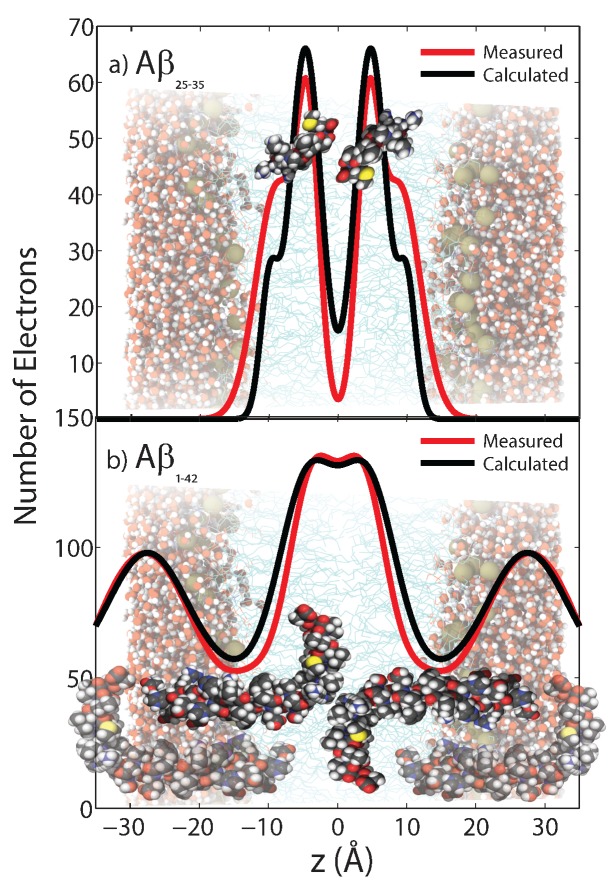
Measured and calculated electron distributionof the membrane-embedded A$\beta_{25-35}$ (**a**) and A$\beta_{1-42}$ (**b**) peptides and their position in the membrane. Good agreement between calculations and experiments was obtained for a position of A$\beta_{25-35}$ in the hydrocarbon membrane core. The peptide takes a slightly tilted orientation, in agreement with computer simulations. The full length A$\beta_{1-42}$ peptide was also found to embed in anionic lipid membranes. These results exclude a membrane-spanning β-sheet structure for Aβ monomers, as was reported from Molecular Dynamics simulations [51,54,55]. (Adapted from [49], copyright PLoS ONE, 2014.)

While Aβ peptides are frequently reported in an extracellular location, A$\beta_{1-40}$ and A$\beta_{1-42}$ molecules were found to strongly interact with negatively charged lipids and to bind to anionic, negatively charged membranes [30,42,43,44,45,46,47], orienting parallel to the membrane surface. Through X-ray and neutron diffraction, Mason et al. [48], Dies et al. [49] and Dante, Hauß and Dencher [9,10,50] observed embedded states for A$\beta_{1-42}$ and the A$\beta_{25-35}$ segment in anionic lipid membranes. Evidence for a membrane-embedded state of the A$\beta_{1-42}$ peptide was first presented by Dante et al. [50]. A high-resolution structure of the embedded states was then presented later by Dies et al. [49]. Both peptides were found to embed as α-helical monomers at low peptide concentrations of 3 mol % [49,51]. The position of the two peptides in anionic lipid bilayers is shown in Figure 5. Barrett et al. recently determined the position of A$\beta_{22-40}$ and A$\beta_{1-42}$ in anionic membranes with and without cholesterol [52]. They presented experimental evidence that the full-length peptide embeds into the membrane, and the peptide fragment occupies two positions [51]—on the membrane surface and embedded into the membrane core. The presence of Aβ peptides in the membranes was also reported to affect the diffusion of the membrane constituents [53].

By probing the membrane structure as a function of Aβ concentration, Aβ was found to localize in three phases associated with the membrane: (1) in the water layer; (2) membrane-bound; or (3) membrane-inserted with a high favorability for the latter [49]. Increasing A$\beta_{25-35}$ concentrations within the membrane increased both the mean tilt of lipid bilayers, and membrane curvature in anionic lipids [21]. The formation of peptide aggregates was found to induce local distortions in the lipid bilayer. While the average membrane thickness was not affected, the distance between acyl chains and the area per tail and tail volume continuously decrease with increasing peptide concentration while the disorder in tail packing increases. The membrane orientation parameter, *f*, was found to decrease and lipid tilt angles increase, indicating an increasing distortion of the bilayers with increasing peptides concentration. The corresponding values are listed in Table 1.

The key features are that (1) the most prominent feature of Aβ are the repeating cross-β sheets which (2) can be parallel or anti-parallel and (3) run “in-register” which suggests residues align with the same residue when placed on top of each other (as depicted in Figure 4).

Both the A$\beta_{1-42}$ and A$\beta_{25-35}$ can have neurotoxic effects, and can independently form β-sheets by hydrogen bonding between parallel polar residues either within the protein, such as in A$\beta_{1-42}$, or with other homologous proteins, such as in A$\beta_{25-35}$ [56]. The formation of these β-sheets is more favorable, with a lower free energy (ΔG), within a hydrophobic surface as provided by the lipid bilayer in a cell membrane.

Next, we will briefly review the role of hydrophobicity in A$\beta_{25-35}$ in terms of membrane attraction and amyloid formation. In particular, the importance of highly resolved physical studies with model systems, such as X-ray diffraction, Molecular Dynamics (MD) simulations, atomic-force microscopy and surface anisotropy, will be paired with biochemical testing to explore a physiologically relevant yet mechanistically sound model for the fragment. The membrane can provide the necessary environment for A$\beta_{25-35}$ to form into cross-β sheets which may initiate cell apoptosis in physiological systems.

## 4. Role of Hydrophobicity in Membrane Incorporation

A$\beta_{1-42}$ contains two largely hydrophobic β-strand segments from residues 9–17 and 27–35 that are connected by a turn segment, giving the final conformation of the peptide as β-turn-β [40]. Not surprisingly, the individual hydrophobic segment A$\beta_{25-35}$ can form this similar cross-β structure independently, and is necessary for the β conformation of the longer A$\beta_{1-42}$ [57,58].

The lipid bilayer provides a unique environment to facilitate this conformation of peptides into β-sheets, which are much more susceptible to the protein’s environment than α-helices, which rely more heavily on the amino acid sequence. Ideally, β-sheets are connected by nonlocal hydrogen bonds, which can be faciliated by an external framework. Pomès and colleagues conducted all-atom MD simulations of truncated peptides in a membrane bilayer to elucidate the mechanisms by which the membrane can promote the formation of β-sheets [59]. In summary, the nonpolar side chains can partition into the hydrophobic phase, leaving the peptide backbone lying in the interface, which promotes the peptide to adopt a β-prone conformation while inducing a partial dehydration of the backbone. As a result, the formation of intra- and intermolecular peptide–peptide hydrogen bonds are favored in the two-dimensional axis of the membrane, i.e., beside each other, rather than with three dimensions of motional freedom. The formation of oligomers in lipid bilayers made of dipalmitoylphosphatidylcholine (DPPC) and cholesterol has been reported in very long μs MD simulations [60], including the formation of short segments of β-sheets between neighboring peptide chains. Interpeptide interactions and membrane perturbation were investigated by Brown and Bevan in atomistic MD simulations [61]. The authors showed the formation of tetramers consisting of four A$\beta_{1-42}$ peptides and a significant increase in β-strand formation. Tetramers were found to perturb POPC bilayers leading to more ordered, rigid membranes. There is evidence that the membrane plays an important role as the interaction between A$\beta_{1-40}$ peptides was reported to depend on lipid composition [62]. The tendency to form dimers was observed to be different in bilayers made of dipalmitoylphosphatidylcholine, POPC, palmitoyloleoylphosphatidylserine (POPS), an equimolar mixture of POPC and palmitoyloleoylethanolamine (POPE), and lipid rafts composed of a 1:1:1 molar ratio of POPC/palmitoylsphingomyelin (PSM)/cholesterol and raft membranes containing ganglioside GM1. Also from MD simulations, the stability and morphology of the oligomers was found to be influenced by hydrophobic and hydrophilic interactions and as such were sensitive to the presence of metal ions, such as Cu${}^{2+}$ and Fe${}^{2+}$ ions [63].

Of the eleven residue fragments in A$\beta_{25-35}$, eight residues are hydrophobic under physiological conditions, which prefer to localize in the tails of the lipid membrane. The Ala-Ile-Ile-Gly-Lys-Met residue near the C-terminus of the peptide fragment tends to be fully incorporated into the lipid tails to minimize electrostatic free energy [64]. Cuco et al. suggest that the interactions between the fragment A$\beta_{25-35}$ and model membranes occur in three segmented stages: adsorption, nucleation, and penetration, supported by MD simulations [65].

In the adsorption phase, small oligomers interact with the polar head groups and induce a surface pressure which increases the area-per-lipid gradually. At low concentrations of A$\beta_{25-35}$, small changes in peptide insertion do not affect area-per-lipid. At high concentrations, however, the area-per-lipid increases at constant surface pressure suggesting that a critical concentration of small oligomers is required for insertion of the peptide into the membrane rather than spontaneous insertion of individual peptides [65]. The full A$\beta_{1-40/42}$ has a partial negative charge at physiological pH which would suggest repulsion from a non-polar negative surface; however, the A$\beta_{1-40/42}$ is still shown to adsorb over time which suggests electrostatic interactions dominate membrane interaction [66,67]. The theoretical isoelectric point of A$\beta_{25-35}$ is 8.75, which suggests a positive partial charge at physiological pH suggesting electrostatic attraction with negatively charged lipid heads [68].

The relative ratio of the membrane-bound and inserted A$\beta_{25-35}$ is based on the membrane’s fluidity and head group charge, which will be explored later in this review [10]. Tsai et al. performed all-atom MD simulations with A$\beta_{25-35}$ in a water-membrane explicit environment and showed that residues 31–35 spontaneously insert into the membrane and “drag” the fragment. The root-mean-squared-displacement of the membrane lipids was found lowest in both the fully inserted phase and the fully expelled phase. The probability mass function (PMF) is greatest when the hydrophobic residues 31–34 are in the head–tail interface [51].

In simulations of the fully transmembrane A$\beta_{16-35}$ in DMPC and POPC micelles, the probability for membrane insertion increases from residue 27 to 40, which encases A$\beta_{25-35}$, and shows strong contact with lipophilic probes [69]. After the A$\beta_{25-35}$ is inserted, the N-terminus shows a 3-fold greater root-mean-square fluctuation, i.e., higher motion, and instability external to the bilayer with the inserted portion [70]. This step entails a large free energy of activation ($\Delta G_{A}$), and is considered reversible [71].

The combination of an electrostatic attraction to the surface and high thermal instability of protruding residues gives rise to the nucleation of the A$\beta_{25-35}$ fragment. Although aggregation is kinetically favorable and occurs over longer timescales, deletion of Met35 reduces time to aggregation in solution as shown by Congo Red staining [32]. Poojari and Strodel investigated tetramer formation in POPC bilayers of different A$\beta_{1-42}$ mutations using MD simulations [72] and showed that peptide-peptide and also peptide-membrane interactions crucially depend on the specific residues.

In essence, the membrane allows the adsorption of Aβ into the membrane which leads to (1) an increase in local concentration for aggregation; the (2) loss of orientational; and (3) conformational freedom in the water-hydrophobic interphase that promotes the formation of amphiphatic β-sheets. Together, the lipid bilayer reduces the Gibbs energy for aggregation by providing the Aβ peptide an environment which reduces the entropy of the aggregated peptide.

## 5. Influence of Intrinsic Membrane Properties on Aβ

Aβ has been found to interact with a diverse range of membrane constituents. The properties of a protein-depleted lipid bilayer can be deduced from the phospholipid ratio [73], presence of cholesterol [74,75,76], and the ionic charge ratio [77]. Using these relations, model bilayers have been generated to perturb the residual properties of A$\beta_{25-35}$ conformation in bilayers as a function of properties [29]. Here, we will review the effect of extrinsic factors on the cell membrane, and the resultant modulated membrane property which gives rise to increased rates of aggregation.

As mentioned, model membranes can be uni- or multi-component and artificially exaggerated to determine the resulting effects on Aβ. DMPC is the best characterized model lipid due to its accessible phase transition behavior between a gel and fluid phase structure offering high structural resolution. DMPC has been the standard model membrane system for many years to mimic physiological systems. However, the two acyl tails in DMPC are saturated, whereas cell membranes consist of a homogeny of saturation and unsaturation in physiological systems. For this reason, researchers have been using POPC as a better model for erythrocyte membranes due to its intrinsic fluidity and resemblance of neuronal membranes. DMPG is a negatively charged phospholipid which allows for modification of lipid surface charges. DMPS is an anionic phospholipid which mimics fluid behavior and is used for doping of other multi-component membrane systems.

### 5.1. Location in the Bilayer

All cell membranes have an intrinsic electron density profile along the bilayer axis with densely charged head groups sandwiching acyl tails of low charge density which become modulated upon peptide incorporation. Using this relation, early neutron diffraction studies showed A$\beta_{25-35}$ inserted deeply into lipid bilayers which is the precursor to channel formation [78]. From X-ray diffraction of stacked lipid bilayers, A$\beta_{25-35}$ was found to localize in either the lipid head group or membrane core state whereas the larger Aβ1-42 was found to align with the bilayer normal [21,49]. All-atom MD simulations show there is a steady-state equilibrium reaching toward full membrane incorporation of the peptide within 3–6 ns. When A$\beta_{25-35}$ conforms within the core, there is a coordination of three hydrogen bonds between the residues and choline head groups within the peptide which becomes stabilized by the malleable neighboring lipid tails [55]. Building upon this, umbrella simulations show the peptide in the membrane-embedded system with a high partitioning coefficient in model membranes, with two free-energy wells in the head groups and especially between Lys28 and the acyl tail [55,69,79]. This signifies that the highest chemical stability for the peptide is within the core group.

### 5.2. Membrane Fluidity and Cholesterol

Physiological concentrations of cholesterol have been shown to play a significant role in the interactions of the Aβ peptide with membranes [10,49,52,80,81,82]. Several studies have made correlations between high cholesterol concentrations and increased risk of Alzheimer’s disease [83,84]. Although cholesterol’s relationship with Alzheimer’s disease may be through cellular signaling, cholesterol affects an intrinsic property of lipid bilayers, membrane fluidity, which in gist encompasses the lateral motion of constituent phospholipids with implications in raft formation, permeability, and stiffness of membranes [85,86,87]. A highly fluid membrane would be composed of well-spaced lipids with a higher degree of freedom in acyl tail motion, whereas a less fluid (or gel) membrane would be tightly packed. Current evidence suggests cholesterol-enriched membranes exist in a homogeneity of fluid and gel phase domains, respectively, [74,76], which creates the possibility of favorable interaction of ligands with a distinct phase [49].

Researchers have used cholesterol-enriched bilayers to generate a dose-dependent relation between fluidity and A$\beta_{25-35}$. Clinical results show higher cholesterol levels can be correlated with greater neural atrophy, and there is strong evidence that this relation is due to the varying bilayer fluidity [83,84,88]. Cholesterol preferentially localizes within the bilayer, which reduces the accessible volume for A$\beta_{25-35}$ interaction in the cholesterol-enriched gel phase [10]. However, this increases the partitioning coefficient for the A$\beta_{25-35}$ into the fluid phase domains [49,89].

Further, MD simulations and X-ray diffraction have shown that gel-phase membranes coordinate hydrogen bonds between residues in the peptide to orient the terminal Met residue externally [49,55]. Due to the localized charge conjugation, this provides an attractive environment for further A$\beta_{25-35}$ residues to localize based solely on electrostatic interactions, producing the precursor to neurotoxic aggregates. Another effector of fluidity is the saturation of the lipid bilayers, as unsaturated bilayers have been experimentally determined to be more fluid than saturated bilayers [90]. A$\beta_{25-35}$ spontaneously forms cross-β sheets in unsaturated bilayers, which supports the notion that there is a free-energy minimum conformation within more fluid bilayers. Further, fluorescence quenching experiments show that A$\beta_{25-35}$ can increase membrane fluidity independently and hypothesize new clinical interventions can use this as a loci of interaction to reduce A$\beta_{25-35}$ induced apoptosis [91].

### 5.3. Metal Ions and Charge Density

It is well known that APPs bind copper (Cu${}^{2+}$) and zinc (Zn${}^{2+}$), and are therefore metalloproteins. The fragments are also influenced by local concentrations of metal ions [92]. The differential in ion concentration, specifically of sodium and potassium ions, is crucial for proper neural signalling and basal function. Ultimately, the neurodegenerative effects of Aβ plaques are a result of ion imbalances and the proliferation of radical oxidative species; however, the electrochemical consequences of these species results in membrane modulation [93].

In physiological membranes, metal cations are also present in the lipid bilayer system which modulate the charge distribution in the head group region affecting bilayer rigidity [94], phase changes [95], lipid clustering [96,97], surface charge, and hydration kinetics [93]. Common metal cations, such as Mn${}^{2+}$, Cu${}^{2+}$, Zn${}^{2+}$, and Ca${}^{2+}$, are found in neural membranes in a heterogeneity between the two leaflets. Other heavier metals such as lead, cadmium, mercury, and arsenic were not found to be significantly different in the progression of Alzheimer’s disease [98]. Increased concentrations of Zn${}^{2+}$ and Cu${}^{2+}$ are observed in plaques, which in conjunction with an exposed Met35 induce oxidative stress on cells [99]. Also, the dysregulation of $Ca^{2+}$ can lead to neuronal cell death [100].

### 5.4. Membrane Curvature

Any embedded peptide in the lipid bilayer can form local distortions surrounding the peptide itself based on the interactions with neighboring phospholipids [101]. Local distortions cause a change in the membrane interface, and thus affect the energy barriers associated with the aggregation of membrane-bound or inserted Aβ.

Imagine a bucket half-filled with water, and place a number floating disks on the surface. If the surface and the disks do not interact, then we expect the disks to simply diffuse based on Brownian or random motion and form an equidistant lattice with respect to one another. However, if a small distortion is made around the edge of the disk, i.e., induces negative curvature around each disk, the disks will begin to come together to minimize the total surface tension of the system. Likewise, if Aβ peptides can induce the bending of neighboring lipids in the membrane interface, measures of aggregation kinetics will be affected as a consequence. For this reason, inhibiting negative membrane curvature is a potential target in anti-Alzheimer’s treatments.

The bending of a monolayer will arise due to the local deformation, but as identified by Pomès [59], the most dominant energy cost is associated with peptide inclusion into the membrane. Hydrophobic mismatch occurs when the hydrophobic region of the peptide is larger, or smaller, than the bilayer hydrophobic thickness, which causes each monolayer leaflet to distort in order to ensure the entire hydrophobic region of the peptide is contained within the hydrophobic core. These local membrane distortions are result of long-range interactions between peptides.

The free energy per amphiphile of a monolayer can be written as [101]:

(2) $$f\left(u,\alpha_{L}\right)=\gamma a_{L}+G\left(u\right)+K\left(\alpha_{L}\right){\left(\nabla^{2}u-\kappa \left(a_{L}\right)\right)}^{2}$$

where γ is the surface tension between the aqueous media and the hydrophobic amphiphile tails, and G(u), a compression–expansion term of the amphiphiles. The thickness of the membrane, *u*, and the area per amphiphile molecule, $a_{L}$, are functions of the distance, *r*, with respect to the inclusion, i.e., u(r) and $a_{L}\left(r\right)$, and are related by an incompressibility condition to keep the lipid volume constant. The other terms stem from bending of the monolayer indicated by the local monolayer curvature $\nabla^{2}u\left(r\right)$. $K(a_{L})$ is the bending stiffness per molecule, so that $K\left(a_{L}\right){\left(\nabla^{2}u\right)}^{2}$ represents the energy related to bending the leaflet. The last term corresponds to the spontaneous curvature of the monolayer, where $\kappa (a_{L})$ is the spontaneous curvature per molecule. The spontaneous curvature mainly depends on structural parameters, such as the composition of the membrane. It plays, however, an important role for the magnitude and the character of the lipid mediated interaction.

**Figure 6:**
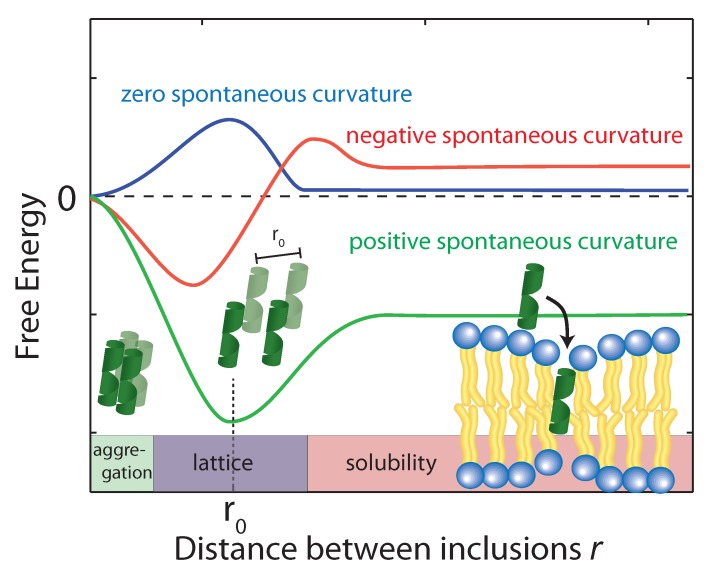
Schematics of the free energy profiles of lipid monolayers for lipids with zero, positive, and negative spontaneous curvature as function of the distance between inclusions. (Adapted from [21], copyright Royal Society of Chemistry, 2016.)

Using the above equation, the membrane perturbation profile and the membrane-induced interactions between an array of inclusions embedded in a two-dimensional membrane have been calculated [102,103,104], and are sketched in Figure 6. In the case of small or vanishing spontaneous curvature, the global energy minimum is obtained at r=0, which favors aggregation. A metastable, dispersed state exists, separated from the aggregated state by an energy barrier.

Aggregation becomes unfavorable for nonzero spontaneous curvature (positive or negative) and the energy becomes minimal at a finite spacing ($r_{0}$) between inclusions [101]. In this state the peptides are expected to arrange on a regular lattice, as for instance observed in the case of purple membrane [105]. The energy at r→∞ is a measure of the energy related to insertion of the peptide into the bilayer. If this energy is negative peptides spontaneously embed in the bilayers. In the case of A$\beta_{25-35}$, the peptide is shorter than the bilayer thickness, such that a positive spontaneous curvature favors peptide insertion.

## 6. Membrane Disruption

The two different mechanisms that have been proposed for membrane disruption of Aβ include the formation of ion-channel like pores or membrane fragmentation [100,106,107]. The biological membrane provides the framework for the initiation of these processes by promoting the formation of cross-β sheets within the membrane [108]. Here, we will briefly review the steps for the formation of these sheets within the membrane.

Amyloid fibrils of A$\beta_{1-40}$ and A$\beta_{1-42}$ form parallel cross-β sheets in solution. Early structural studies of A$\beta_{25-35}$ from NMR suggest that residues 25–27 are naturally disordered, and FTIR shows residues 26–33 are in random coil or α-helix [109,110]. Circular dichroism studies concluded that both increased peptide concentration and time of solvation increased the propensity for residues 25–35 to form cross-β sheets [111,112]. As aforementioned, A$\beta_{25-35}$ can exist in two states either perpendicular to the bilayer in the head groups, and internally aligned roughly parallel with the acyl tail [49]. At low concentrations of A$\beta_{25-35}$ forms α-helical monomers, but at higher concentrations, these peptides can begin to aggregate into antiparallel cross-β sheets [21]. This can be explained by spontaneous population shifts between the two states, increased membrane fluidity, and the charge distribution in an A$\beta_{25-35}$-enriched membrane.

If the A$\beta_{25-35}$ is being exchanged between two states, a chemical steady-state is formed in the following form:

(3) $$A\beta_{Head}<=>\left[k_{\alpha }\right]\left[k_{-\alpha }\right]A\beta_{Tail}<=>\left[k_{\beta }\right]A\beta_{\beta -Sheet}$$

where the rate constants $k_{\alpha }$, $k_{-\alpha }$, and $k_{\beta }$ correspond to insertion, expulsion, and conformation respectively. Indirect measurements for these values have been calculated from fluorescence, and computer simulations, but as peptide concentration increases, a greater number of individual peptides would be in the inserted state [49,113,114].

When more peptides are in the inserted phase, steric interactions and hydrogen bonding between neighboring peptides in a “steric zipper” [2] for which interactions are mutually orthogonal to the electrostatic attraction between lipids in a bilayer. Specifically, the glutamine and asparagine residues can form hydrogen bonds with to the identical strands independent of whether the cross-β sheet is parallel or anti-parallel. This explanation is sufficient to describe why A$\beta_{25-35}$ cannot easily localize into cholesterol-enriched membranes, where the lateral forces are quite significant deterring the formation of aggregate.

**Figure 7:**
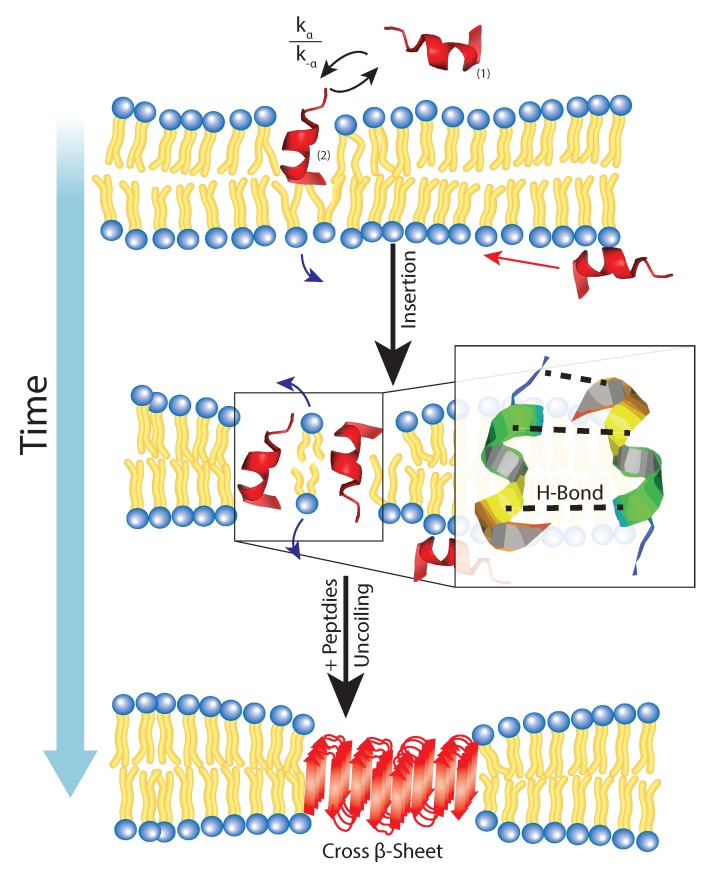
A$\beta_{25-35}$ coexists in an external and inserted phase in which there is a certain free energy barrier which inhibits full insertion. For this reason, there is a rate of insertion and expulsion within the membrane. After the insertion, H-bonding between enighbouring Glu and Arg residues coordinate lateral attraction and promote the formation of a cross-β sheet through the U-turn N-terminus residues external to the bilayer.

All in all, the full mechanism for membrane-mediated Aβ remains elusive; however, from current understanding of Aβ fragments and their influence on the membrane provide a framework for the processes involved in aggregation. As depicted in Figure 7, the bilayer offers a site of high stability for A$\beta_{25-35}$ monomers allowing some rate constant of adsorption and insertion $k_{\alpha }$ and of course, some rate constant of expulsion $k_{-\alpha }$. From this, the stabilization of this inserted form allows neighboring peptides to coordinate hydrogen-bonding, and long-range lipid attractions to minimize membrane surface tension between Aβ fragments. This allows for folding and uncoiling of the peptide to form more stable cross-β sheets which makes the membrane a key role in the nucleation of these aggregates.

## 7. Conclusions

At the molecular level, Aβ aggregation remains a highly debated and complex phenomenon. Recent work has begun to reveal high-resolution structural dynamics of the formation of Aβ aggregates in Alzheimer’s disease; however, we also see that the presence of a membrane support can mediate the formation of aggregates and molecular cross-β sheets. The presence of a membrane can stabilize the the lateral hydrogen bonding in A$\beta_{1-42}$ and A$\beta_{25-35}$ to reduce the free energy of oligomerization. Thus, factors that influence the membrane affect the energy of this transition, such as membrane curvature, surface charge, and hydrodynamic diameter. In summary, experiments from model membrane systems with Aβ show that the environment plays a major role in the pathogenesis of Alzheimer’s disease. 

## Figures and Tables

**Table 1:** Structural membrane parameters for the different amyloid $\beta_{25-35}$ concentrations. While the lamellar $d_{z}$-spacing changes with peptide concentration, the head group-head group distance $d_{HH}$ is constant such that changes can be attributed to changes in the water layer thickness, $d_{water}$. Distance between acyl chains ($a_{T}$), area per tail ($A_{T}$) and tail volume ($V_{T}$) continuously decrease with increasing peptide concentration while the disorder in tail packing increases ($\Delta a_{T}$). The membrane orientation parameter, *f*, decreases and lipid tilt angles increase, indicating and increasing distortion and bending of the bilayers with increasing peptides concentration. (Values from [21].)

A$\beta_{25-35}$ (mol%)	$d_{z}$ (Å)	$d_{HH}$ (Å)	$d_{water}$ (Å)	$a_{T}$ (Å)	$\Delta a_{T}$ (Å)	$A_{T}$ (Å${}^{2}$)	$V_{T}$ (Å${}^{3}$)	f Membranes	Lipid Tail Tilt (${}^{\circ }$)
0	59.0 ± 0.1	39.4 ± 1.0	19.6 ± 0.5	5.20 ± 0.05	0.59 ± 0.02	23.4 ± 0.1	922 ± 1	0.96 ± 0.02	19.2 ± 5
3	54.9 ± 0.1	39.1 ± 1.0	15.8 ± 0.4	5.21 ± 0.05	0.61 ± 0.02	23.5 ± 0.1	919 ± 1	0.92 ± 0.03	21.4 ± 2.3
10	61.6 ± 0.4	39.2 ± 1.0	22.4 ± 0.6	5.21 ± 0.05	0.67 ± 0.02	23.5 ± 0.1	921 ± 1	0.90 ± 0.03	20.5 ± 2.3
20	58.0 ± 0.2	39.3 ± 1.0	18.7 ± 0.5	5.06 ± 0.05	0.74 ± 0.02	22.6 ± 0.1	886 ± 1	0.86 ± 0.03	25.4 ± 3

## References

[1] Hardy J., Selkoe D.J. (2002). The amyloid hypothesis of Alzheimer’s disease: Progress and problems on the road to therapeutics. Science.

[2] Eisenberg D., Jucker M. (2012). The amyloid state of proteins in human diseases. Cell.

[3] Goedert M. (2015). Alzheimer’s and Parkinson’s diseases: The prion concept in relation to assembled A*β*, tau, and *α*-synuclein. Science.

[4] Nasica-Labouze J., Nguyen P.H., Sterpone F., Berthoumieu O., Buchete N.V., Coté S., Simone A.D., Doig A.J., Faller P., Garcia A. (2015). Amyloid *β* protein and Alzheimer’s disease: When computer simulations complement experimental studies. Chem. Rev..

[5] Knowles T.P., Vendruscolo M., Dobson C.M. (2014). The amyloid state and its association with protein misfolding diseases. Nat. Rev. Mol. Cell Biol..

[6] Nelson R., Sawaya M.R., Balbirnie M., Madsen A.Ø., Riekel C., Grothe R., Eisenberg D. (2005). Structure of the cross-*β* spine of amyloid-like fibrils. Nature.

[7] Mattson M.P. (1997). Cellular actions of beta-amyloid precursor protein and its soluble and fibrillogenic derivatives. Physiol. Rev..

[8] Hamley I. (2012). The amyloid beta peptide: A chemist’s perspective. Role in Alzheimer’s and fibrillization. Chem. Rev..

[9] Dante S., Hauss T., Dencher N.A. (2002). *β*-Amyloid 25 to 35 Is Intercalated in Anionic and Zwitterionic Lipid Membranes to Different Extents. Biophys. J..

[10] Dante S., Hauß T., Dencher N.A. (2006). Cholesterol inhibits the insertion of the Alzheimer’s peptide A*β* (25–35) in lipid bilayers. Eur. Biophys. J..

[11] Haass C., Selkoe D.J. (2007). Soluble protein oligomers in neurodegeneration: Lessons from the Alzheimer’s amyloid *β*-peptide. Nat. Rev. Mol. Cell Biol..

[12] Ladiwala A.R.A., Litt J., Kane R.S., Aucoin D.S., Smith S.O., Ranjan S., Davis J., Van Nostrand W.E., Tessier P.M. (2012). Conformational differences between two amyloid *β* oligomers of similar size and dissimilar toxicity. J. Biol. Chem..

[13] Breydo L., Kurouski D., Rasool S., Milton S., Wu J.W., Uversky V.N., Lednev I.K., Glabe C.G. (2016). Structural differences between amyloid beta oligomers. Biochem. Biophys. Res. Commun..

[14] Krstic D., Knuesel I. (2013). Deciphering the mechanism underlying late-onset Alzheimer disease. Nat. Rev. Neurol..

[15] Drachman D.A. (2014). The amyloid hypothesis, time to move on: Amyloid is the downstream result, not cause, of Alzheimer’s disease. Alzheimer’s Dement..

[16] Serra-Batiste M., Ninot-Pedrosa M., Bayoumi M., Gairí M., Maglia G., Carulla N. (2016). A*β*42 assembles into specific *β*-barrel pore-forming oligomers in membrane-mimicking environments. Proc. Natl. Acad. Sci. USA.

[17] Spires-Jones T.L., Hyman B.T. (2014). The intersection of amyloid beta and tau at synapses in Alzheimer’s disease. Neuron.

[18] Nicoll J.A., Wilkinson D., Holmes C., Steart P., Markham H., Weller R.O. (2003). Neuropathology of human Alzheimer disease after immunization with amyloid-*β* peptide: A case report. Nat. Med..

[19] Drolle E., Negoda A., Hammond K., Pavlov E., Leonenko Z. (2017). Changes in lipid membranes may trigger amyloid toxicity in Alzheimer’s disease. arXiv Preprint.

[20] Ahmed M., Davis J., Aucoin D., Sato T., Ahuja S., Aimoto S., Elliott J.I., Van Nostrand W.E., Smith S.O. (2010). Structural conversion of neurotoxic amyloid-*β*_1-42_ oligomers to fibrils. Nat. Struct. Mol. Biol..

[21] Tang J., Alsop R.J., Backholm M., Dies H., Shi A.C., Rheinstädter M.C. (2016). Amyloid-*β* 25–35 peptides aggregate into cross-*β* sheets in unsaturated anionic lipid membranes at high peptide concentrations. Soft Matter.

[22] Hardy J.A., Higgins G.A. (1992). Alzheimer’s disease: The amyloid cascade hypothesis. Science.

[23] Ono K., Condron M.M., Teplow D.B. (2009). Structure–neurotoxicity relationships of amyloid *β*-protein oligomers. Proc. Natl. Acad. Sci. USA.

[24] Fisher Y., Nemirovsky A., Baron R., Monsonego A. (2010). T cells specifically targeted to amyloid plaques enhance plaque clearance in a mouse model of Alzheimer’s disease. PLoS ONE.

[25] Chen W.T., Hong C.J., Lin Y.T., Chang W.H., Huang H.T., Liao J.Y., Chang Y.J., Hsieh Y.F., Cheng C.Y., Liu H.C. (2012). Amyloid-beta (A*β*) D7H mutation increases oligomeric A*β*42 and alters properties of A*β*-zinc/copper assemblies. PLoS ONE.

[26] Hane F., Attwood S., Leonenko Z. (2014). Comparison of three competing dynamic force spectroscopy models to study binding forces of amyloid-*β* (1–42). Soft Matter.

[27] Tomaselli S., Esposito V., Vangone P., van Nuland N.A., Bonvin A.M., Guerrini R., Tancredi T., Temussi P.A., Picone D. (2006). The *α*-to-*β* Conformational Transition of Alzheimer’s A*β*-(1–42) Peptide in Aqueous Media is Reversible: A Step by Step Conformational Analysis Suggests the Location of *β* Conformation Seeding. ChemBioChem.

[28] D’Ursi A.M., Armenante M.R., Guerrini R., Salvadori S., Sorrentino G., Picone D. (2004). Solution Structure of Amyloid *β*-Peptide (25–35) in Different Media. J. Med. Chem..

[29] Pabst G., Kučerka N., Nieh M.P., Rheinstädter M., Katsaras J. (2010). Applications of neutron and X-ray scattering to the study of biologically relevant model membranes. Chem. Phys. Lipids.

[30] Hane F., Drolle E., Gaikwad R., Faught E., Leonenko Z. (2011). Amyloid-*β* aggregation on model lipid membranes: An atomic force microscopy study. J. Alzheimer’s Dis..

[31] Forloni G., Chiesa R., Smiroldo S., Verga L., Salmona M., Tagliavini F., Angeretti N. (1993). Apoptosis mediated neurotoxicity induced by chronic application of *β* amyloid fragment 25–35. Neuroreport.

[32] Millucci L., Ghezzi L., Bernardini G., Santucci A. (2010). Conformations and biological activities of amyloid beta peptide 25–35. Curr. Protein Pept. Sci..

[33] Walsh D., Klyubin I., Fadeeva J., Rowan M., Selkoe D. (2002). Amyloid-*β* oligomers: Their production, toxicity and therapeutic inhibition. Biochem. Soc. Trans..

[34] Bokvist M., Lindström F., Watts A., Gröbner G. (2004). Two types of Alzheimer’s *β*-amyloid (1–40) peptide membrane interactions: Aggregation preventing transmembrane anchoring versus accelerated surface fibril formation. J. Mol. Biol..

[35] Cohen A.S., Calkins E. (1959). Electron microscopic observations on a fibrous component in amyloid of diverse origins. Nature.

[36] Eanes E., Glenner G. (1968). X-ray diffraction studies on amyloid filaments. J. Histochem. Cytochem..

[37] Colvin M.T., Silvers R., Ni Q.Z., Can T.V., Sergeyev I., Rosay M., Donovan K.J., Michael B., Wall J., Linse S. (2016). Atomic resolution structure of monomorphic A*β*42 amyloid fibrils. J. Am. Chem. Soc..

[38] Lashuel H.A., LaBrenz S.R., Woo L., Serpell L.C., Kelly J.W. (2000). Protofilaments, filaments, ribbons, and fibrils from peptidomimetic self-assembly: Implications for amyloid fibril formation and materials science. J. Am. Chem. Soc..

[39] Makin O.S., Serpell L.C. (2005). Structures for amyloid fibrils. FEBS J..

[40] Lührs T., Ritter C., Adrian M., Riek-Loher D., Bohrmann B., Döbeli H., Schubert D., Riek R. (2005). 3D structure of Alzheimer’s amyloid-*β* (1–42) fibrils. Proc. Natl. Acad. Sci. USA.

[41] Gazit E. (2002). A possible role for *π*-stacking in the self-assembly of amyloid fibrils. FASEB J..

[42] Del Mar Martínez-Senac M., Villalaín J., Gómez-Fernández J.C. (1999). Structure of the Alzheimer *β*-amyloid peptide (25–35) and its interaction with negatively charged phospholipid vesicles. Eur. J. Biochem..

[43] Maltseva E., Brezesinski G. (2004). Adsorption of Amyloid *β* (1–40) Peptide to Phosphatidylethanolamine Monolayers. ChemPhysChem.

[44] Thakur G., Micic M., Leblanc R.M. (2009). Surface chemistry of Alzheimer’s disease: A Langmuir monolayer approach. Coll. Surf. B Biointerfaces.

[45] Sani M.A., Gehman J.D., Separovic F. (2011). Lipid matrix plays a role in A*β* fibril kinetics and morphology. FEBS Letters.

[46] Ding H., Schauerte J.A., Steel D.G., Gafni A. (2012). *β*-Amyloid (1–40) Peptide Interactions with Supported Phospholipid Membranes: A Single-Molecule Study. Biophys. J..

[47] Ahyayauch H., Raab M., Busto J.V., Andraka N., Arrondo J.L.R., Masserini M., Tvaroska I., Goni F.M. (2012). Binding of *β*-Amyloid (1–42) Peptide to Negatively Charged Phospholipid Membranes in the Liquid-Ordered State: Modeling and Experimental Studies. Biophys. J..

[48] Mason R.P., Estermyer J.D., Kelly J.F., Mason P.E. (1996). Alzheimer’s disease amyloid *β* peptide 25–35 is localized in the membrane hydrocarbon core: X-ray diffraction analysis. Biochem. Biophys. Res. Commun..

[49] Dies H., Toppozini L., Rheinstädter M.C. (2014). The interaction between amyloid-*β* peptides and anionic lipid membranes containing cholesterol and melatonin. PLoS ONE.

[50] Dante S., Hauss T., Steitz R., Canale C., Dencher N.A. (2011). Nanoscale structural and mechanical effects of *β*-amyloid (1–42) on polymer cushioned membranes: A combined study by neutron reflectometry and {AFM} Force Spectroscopy. BBA Biomembr..

[51] Tsai H.H.G., Lee J.B., Tseng S.S., Pan X.A., Shih Y.C. (2010). Folding and membrane insertion of amyloid-*β* (25–35) peptide and its mutants: Implications for aggregation and neurotoxicity. Proteins Struct. Funct. Bioinform..

[52] Barrett M.A., Alsop R.J., Hauß T., Rheinstädter M.C. (2015). The Position of A*β*22–40 and A*β*1–42 in Anionic Lipid Membranes Containing Cholesterol. Membranes.

[53] Barrett M.A., Trapp M., Lohstroh W., Seydel T., Ollivier J., Ballauff M., Dencher N.A., Hauß T. (2016). Alzheimer’s peptide amyloid-*β*, fragment 22–40, perturbs lipid dynamics. Soft Matter.

[54] Strodel B., Lee J.W., Whittleston C.S., Wales D.J. (2010). Transmembrane structures for Alzheimer’s A*β*1–42 oligomers. J. Am. Chem. Soc..

[55] Poojari C., Kukol A., Strodel B. (2013). How the amyloid-*β* peptide and membranes affect each other: An extensive simulation study. Biochim. Biophys. Acta Biomembr..

[56] Petkova A.T., Leapman R.D., Guo Z., Yau W.M., Mattson M.P., Tycko R. (2005). Self-propagating, molecular-level polymorphism in Alzheimer’s ß-amyloid fibrils. Science.

[57] Liu R., McAllister C., Lyubchenko Y., Sierks M.R. (2004). Residues 17–20 and 30–35 of beta-amyloid play critical roles in aggregation. J. Neurosci. Res..

[58] Millucci L., Raggiaschi R., Franceschini D., Terstappen G., Santucci A. (2009). Rapid aggregation and assembly in aqueous solution of A*β* (25–35) peptide. J. Biosci..

[59] Nikolic A., Baud S., Rauscher S., Pomès R. (2011). Molecular mechanism of *β*-sheet self-organization at water-hydrophobic interfaces. Proteins Struct. Funct. Bioinform..

[60] Zhao L.N., Chiu S.W., Benoit J., Chew L.Y., Mu Y. (2011). Amyloid *β* peptides aggregation in a mixed membrane bilayer: A molecular dynamics study. J. Phys. Chem. B.

[61] Brown A.M., Bevan D.R. (2016). Molecular Dynamics Simulations of Amyloid *β*-Peptide (1–42): Tetramer Formation and Membrane Interactions. Biophys. J..

[62] Lemkul J.A., Bevan D.R. (2013). Aggregation of Alzheimer’s amyloid *β*-peptide in biological membranes: A molecular dynamics study. Biochemistry.

[63] Dorosh L., Stepanova M. (2017). Probing oligomerization of amyloid beta peptide in silico. Mol. BioSyst..

[64] Sarroukh R., Cerf E., Derclaye S., Dufrêne Y.F., Goormaghtigh E., Ruysschaert J.M., Raussens V. (2011). Transformation of amyloid *β* (1–40) oligomers into fibrils is characterized by a major change in secondary structure. Cell. Mol. Life Sci..

[65] Cuco A., Serro A.P., Farinha J.P., Saramago B., da Silva A.G. (2016). Interaction of the Alzheimer A*β* (25–35) peptide segment with model membranes. Coll. Surf. B Biointerfaces.

[66] Giacomelli C.E., Norde W. (2003). Influence of hydrophobic Teflon particles on the structure of amyloid *β*-peptide. Biomacromolecules.

[67] Giacomelli C.E., Norde W. (2005). Conformational Changes of the Amyloid *β*-Peptide (1–40) Adsorbed on Solid Surfaces. Macromol. Biosci..

[68] Zhao H., Tuominen E.K., Kinnunen P.K. (2004). Formation of amyloid fibers triggered by phosphatidylserine-containing membranes. Biochemistry.

[69] Chebaro Y., Mousseau N., Derreumaux P. (2009). Structures and Thermodynamics of Alzheimer’s Amyloid-*β* A*β* (16–35) Monomer and Dimer by Replica Exchange Molecular Dynamics Simulations: Implication for Full-Length A*β* Fibrillation. J. Phys. Chem. B.

[70] Dominguez L., Meredith S.C., Straub J.E., Thirumalai D. (2014). Transmembrane fragment structures of amyloid precursor protein depend on membrane surface curvature. J. Am. Chem. Soc..

[71] Jang H., Arce F.T., Ramachandran S., Kagan B.L., Lal R., Nussinov R. (2014). Disordered amyloidogenic peptides may insert into the membrane and assemble into common cyclic structural motifs. Chem. Soc. Rev..

[72] Poojari C., Strodel B. (2013). Stability of transmembrane amyloid *β*-peptide and membrane integrity tested by molecular modeling of site-specific A*β*42 mutations. PLoS ONE.

[73] Van Meer G., Voelker D.R., Feigenson G.W. (2008). Membrane lipids: Where they are and how they behave. Nat. Rev. Mol. Cell Biol..

[74] Armstrong C.L., Marquardt D., Dies H., Kučerka N., Yamani Z., Harroun T.A., Katsaras J., Shi A.C., Rheinstädter M.C. (2013). The observation of highly ordered domains in membranes with cholesterol. PLoS ONE.

[75] Rheinstädter M.C., Mouritsen O.G. (2013). Small-scale structure in fluid cholesterol—Lipid bilayers. Curr. Opin. Coll. Interface Sci..

[76] Toppozini L., Meinhardt S., Armstrong C.L., Yamani Z., Kučerka N., Schmid F., Rheinstädter M.C. (2014). Structure of cholesterol in lipid rafts. Phys. Rev. Lett..

[77] Düzgünes N., Papahadjopoulos D. (1983). Ionotropic effects on phospholipid membranes: Calcium/magnesium specificity in binding, fluidity and fusion. Membr. Fluidity Biol..

[78] Dante S., Hauss T., Dencher N.A. (2003). Insertion of externally administered amyloid *β* peptide 25–35 and perturbation of lipid bilayers. Biochemistry.

[79] Davis C.H., Berkowitz M.L. (2009). Structure of the Amyloid-*β* (1–42) Monomer Absorbed to Model Phospholipid Bilayers: A Molecular Dynamics Study. J. Phys. Chem. B.

[80] Pappolla M.A., Sos M., Omar R.A., Bick R.J., Hickson-Bick D.L., Reiter R.J., Efthimiopoulos S., Robakis N.K. (1997). Melatonin prevents death of neuroblastoma cells exposed to the Alzheimer amyloid peptide. J. Neurosci..

[81] Drolle E., Gaikwad R.M., Leonenko Z. (2012). Nanoscale electrostatic domains in cholesterol-laden lipid membranes create a target for amyloid binding. Biophys. J..

[82] Hane F., Drolle E., Leonenko Z. (2014). Amyloid-*β* (1–40) restores adhesion properties of pulmonary surfactant, counteracting the effect of cholesterol. Phys. Chem. Chem. Phys..

[83] Fonseca A.C.R., Resende R., Oliveira C.R., Pereira C.M. (2010). Cholesterol and statins in Alzheimer’s disease: Current controversies. Exp. Neurol..

[84] Puglielli L., Tanzi R.E., Kovacs D.M. (2003). Alzheimer’s disease: The cholesterol connection. Nat. Neurosci..

[85] Armstrong C.L., Barrett M.A., Hiess A., Salditt T., Katsaras J., Shi A.C., Rheinstädter M.C. (2012). Effect of cholesterol on the lateral nanoscale dynamics of fluid membranes. Eur. Biophys. J..

[86] Kagawa R., Hirano Y., Taiji M., Yasuoka K., Yasui M. (2013). Dynamic interactions of cations, water and lipids and influence on membrane fluidity. J. Membr. Sci..

[87] Armstrong C.L., Häußler W., Seydel T., Katsaras J., Rheinstädter M.C. (2014). Nanosecond lipid dynamics in membranes containing cholesterol. Soft Matter.

[88] Wood W.G., Eckert G.P., Igbavboa U., Müller W.E. (2003). Amyloid beta-protein interactions with membranes and cholesterol: Causes or casualties of Alzheimer’s disease. BBA Biomembr..

[89] Williams T.L., Serpell L.C. (2011). Membrane and surface interactions of Alzheimer’s A*β* peptide-insights into the mechanism of cytotoxicity. FEBS J..

[90] Armstrong C., Trapp M., Peters J., Seydel T., Rheinstädter M. (2011). Short range ballistic motion in fluid lipid bilayers studied by quasi-elastic neutron scattering. Soft Matter.

[91] Yang X., Sheng W., Sun G.Y., Lee J.C.M. (2011). Effects of fatty acid unsaturation numbers on membrane fluidity and *α*-secretase-dependent amyloid precursor protein processing. Neurochem. Int..

[92] Hane F., Tran G., Attwood S.J., Leonenko Z. (2013). Cu 2+ affects amyloid-*β* (1–42) aggregation by increasing peptide-peptide binding forces. PLoS ONE.

[93] Alsop R.J., Schober R.M., Rheinstädter M.C. (2016). Swelling of phospholipid membranes by divalent metal ions depends on the location of the ions in the bilayers. Soft Matter.

[94] Pabst G., Hodzic A., Štrancar J., Danner S., Rappolt M., Laggner P. (2007). Rigidification of neutral lipid bilayers in the presence of salts. Biophys. J..

[95] Hauser H. (1991). Effect of inorganic cations on phase transitions. Chem. Phys. Lipids.

[96] Schultz Z.D., Pazos I.M., McNeil-Watson F.K., Lewis E.N., Levin I.W. (2009). Magnesium-induced lipid bilayer microdomain reorganizations: Implications for membrane fusion. J. Phys. Chem. B.

[97] Wang Y.H., Collins A., Guo L., Smith-Dupont K.B., Gai F., Svitkina T., Janmey P.A. (2012). Divalent cation-induced cluster formation by polyphosphoinositides in model membranes. J. Am. Chem. Soc..

[98] Park J.H., Lee D.W., Park K.S., Joung H. (2014). Serum trace metal levels in Alzheimer’s disease and normal control groups. Am. J. Alzheimer’s Dis. Other Dement..

[99] Butterfield D.A., Kanski J. (2002). Methionine residue 35 is critical for the oxidative stress and neurotoxic properties of Alzheimer’s amyloid *β*-peptide 1–42. Peptides.

[100] Jang H., Connelly L., Arce F.T., Ramachandran S., Lal R., Kagan B.L., Nussinov R. (2013). Alzheimer’s disease: Which type of amyloid-preventing drug agents to employ?. Phys. Chem. Chem. Phys..

[101] Armstrong C.L., Sandqvist E., Rheinstädter M.C. (2011). Protein-Protein Interactions in Membranes. Protein Pept. Lett..

[102] Aranda-Espinoza H., Berman A., Dan N., Pincus P., Safran S. (1996). Interaction between inclusions embedded in membranes. Biophys. J..

[103] Dan N., Pincus P., Safran S.A. (1993). Membrane-induced interactions between inclusions. Langmuir.

[104] Dan N., Berman A., Pincus P., Safran S.A. (1994). Membrane-induced interactions between inclusions. J. Phys. II.

[105] Rheinstädter M.C., Schmalzl K., Wood K., Strauch D. (2009). Protein-protein interaction in Purple Membrane. Phys. Rev. Lett..

[106] Diaz J.C., Simakova O., Jacobson K.A., Arispe N., Pollard H.B. (2009). Small molecule blockers of the Alzheimer A*β* calcium channel potently protect neurons from A*β* cytotoxicity. Proc. Natl. Acad. Sci. USA.

[107] Jarvet J., Danielsson J., Damberg P., Oleszczuk M., Gräslund A. (2007). Positioning of the Alzheimer A*β* (1–40) peptide in SDS micelles using NMR and paramagnetic probes. J. Biomol. NMR.

[108] Fitzpatrick A.W., Debelouchina G.T., Bayro M.J., Clare D.K., Caporini M.A., Bajaj V.S., Jaroniec C.P., Wang L., Ladizhansky V., Müller S.A. (2013). Atomic structure and hierarchical assembly of a cross-*β* amyloid fibril. Proc. Natl. Acad. Sci. USA.

[109] Halverson K., Fraser P.E., Kirschner D.A., Lansbury P.T. (1990). Molecular determinants of amyloid deposition in Alzheimer’s disease: Conformational studies of synthetic *β*-protein fragments. Biochemistry.

[110] Kohno T., Kobayashi K., Maeda T., Sato K., Takashima A. (1996). Three-Dimensional Structures of the Amyloid *β* Peptide (25–35) in Membrane-Mimicking Environment. Biochemistry.

[111] Terzi E., Hoelzemann G., Seelig J. (1994). Reversible Random Coil-*β*-Sheet Transition of the Alzheimer *β*-Amyloid Fragment (25–35). Biochemistry.

[112] El-Agnaf O., Guthrie D.J., Walsh D.M., Irvine G.B. (1998). The influence of the central region containing residues 19–25 on the aggregation properties and secondary structure of Alzheimer’s *β*-amyloid peptide. Eur. J. Biochem..

[113] Di Scala C., Chahinian H., Yahi N., Garmy N., Fantini J. (2014). Interaction of Alzheimer’s *β*-amyloid peptides with cholesterol: Mechanistic insights into amyloid pore formation. Biochemistry.

[114] Bachmann M. (2014). Thermodynamics and Statistical Mechanics of Macromolecular Systems.

